# Timely antiretroviral prophylaxis during pregnancy effectively reduces HIV mother-to-child transmission in eight counties in China: a prospective study during 2004–2011

**DOI:** 10.1038/srep34526

**Published:** 2016-10-10

**Authors:** Qian Wang, Linhong Wang, Liwen Fang, Ailing Wang, Xi Jin, Fang Wang, Xiaoyan Wang, Yaping Qiao, Sheena G. Sullivan, Shannon Rutherford, Lei Zhang

**Affiliations:** 1 National Center for women and children’s health, China Center for Disease Control and prevention, Beijing, China; 2 National Center for Chronic and Non-communicable Disease Control and Prevention, China Center for Disease Control and Prevention, Beijing, China; 3 WHO Collaborating Centre for Reference and Research on Influenza, Melbourne, Australia; 4 Affiliation Centre for Environment and Population Health, Griffith University, Australia; 5 Research Center for Public Health, School of Medicine, Tsinghua University, Beijing, China; 6 Central Clinical School, Faculty of Medicine, Nursing and Health Sciences, Monash University, Melbourne, VIC, Australia; 7 School of Public Health and Preventive Medicine, Faculty of Medicine, Nursing and Health Sciences, Monash University, Melbourne, VIC, Australia; 8 Melbourne Sexual Health Centre, Alfred Health, Melbourne, VIC, Australia

## Abstract

This study investigates the improvement of the prevention of mother-to-child transmission (PMTCT) of Human Immunodeficiency Virus (HIV) in China during 2004–2011. A clinic-based prospective study was conducted among HIV-positive pregnant women and their children in eight counties across China. Associated factors of mother-to-child transmission were analyzed using regression analysis. A total of 1,387 HIV+ pregnant women and 1,377 HIV-exposed infants were enrolled. The proportion of pregnant women who received HIV testing increased significantly from 45.1% to 98.9% during 2004–2011. Among whom, the proportion that received antiretroviral (ARV) prophylaxis increased from 61% to 96%, and the corresponding coverage in children increased from 85% to 97% during the same period. In contrast, single-dose nevirapine treatment during delivery declined substantially from 97.9% to 12.7%. Vertical transmission of HIV declined from 11.1% (95% confidence interval [CI]: 5.7–23.3%) in 2004 to 1.2% (95% CI: 0.1–5.8%) in 2011. Women who had a vaginal delivery (compared to emergency caesarian section (odds ratio [OR] = 0.46; 0.23–0.96)) and mothers on multi-ARVs (OR = 0.11; 0.04–0.29) were less likely to transmit HIV to their newborns. Increasing HIV screening enabled timely HIV care and prophylaxis to reduce vertical transmission of HIV. Early and consistent treatment with multi-ARVs during pregnancy is vital for PMTCT.

In 2013, 3.2 million children under 15 were living with human immunodeficiency virus (HIV) globally, and 240,000 newborns were newly infected with HIV^1^. More than 90% of which were living in sub-Saharan Africa^2^. Mother to child transmission remains the primary mode of HIV infection in children in developing country settings. During the past decades, significant progress has been made in scaling up the prevention of HIV mother-to-child transmission (PMTCT) services to pregnant women, particularly in low- and middle- income countries^3^,^4^. Key PMTCT procedures include early HIV counselling and testing, antiretroviral (ARV) drug use, safe childbirth practices and exclusive formulae-feeding. Without appropriate PMTCT, vertical transmission of HIV can be as high as 15–34%^5^,^6^, but this rate can be substantially reduced to below 2% in the presence of PMTCT programs^7^,^8^. In many developed country settings, vertical transmission of HIV has been nearly eliminated or contained to a very low level (<1%)^6^,^8^,^9^. In 2014, the Joint United Nations Programme on HIV/AIDS put forward an ambitious target to achieve: 90% of HIV-infected people to be diagnosed, 90% of diagnosed individuals to receive treatment, and 90% of diagnosed individuals to have sustained viral suppression^10^ by 2020. Achieving these targets is projected to eliminate HIV by 2030^11^.

China has followed the typical pattern of the Asian HIV epidemic^12^. The epidemic was first initiated among injecting drug users but subsequently spread to other key affected populations. In 2013, an estimated 870,000 people were living with HIV/AIDS in China. Among them, 69.5% were infected due to heterosexual transmission and 22.2% were female^13^, leading to concerns of HIV transmission to their children. The PMTCT program was first piloted in Shangcai county of China in 2001 with the support of United Nations Children’s Fund (UNICEF)^14^. Two years later, the program was expanded to eight cities/counties in five Chinese provinces with significant HIV epidemics. By the end of 2012, 1,156 Chinese counties/districts had been covered by the program^14^.

The national PMTCT guideline in China is based on a four-pronged approach, which recommends a comprehensive integration of routine maternal, newborn, child and reproductive health services at all levels. Provider-initiated testing and counseling (PITC) services were offered free of charge to all pregnant women who attend routine antenatal, delivery and post-natal services in the country, as recommended by the World Health Organization (WHO)^15^. PITC is provided routinely, but attendees can choose to opt out. ARV prophylaxis is provided to infected mothers upon diagnosis and all infants born to HIV-positive mothers. Early infant diagnosis is limited in China^14^. Exposed children receive free HIV antibody screening at 12 or 18 months after birth. These measures have reduced HIV vertical transmission from 8.1% (57/702) in 2009 to 6.7% (145/2180) in 2013^14^. Despite the concerted efforts for scaling up the national PMTCT program in China, information on the PMTCT program is under-represented. Only limited data have been published to document the effects of the implemented program. In particular, information on linkage to HIV care and treatment, timing and coverage of ARV prophylaxis is important to evaluate PMTCT. This study aims to assess the improvements in PMTCT utilization among pregnant women and identify its impacts on the rate of mother-to-child transmission and associated factors. Findings will provide important evidence for future implementation of PMTCT services and inform health policies towards improving HIV prevention and control strategies.

## Method

### Study setting

An institution-based prospective study was carried out among HIV-positive pregnant women and their children. The study was conducted at eight Chinese counties, including Ruili, Longchuan, and Lincang in Yunnan, Shangcai and Weishi in Henan, Hezhou and Lingshan in Guangxi and Yining of Xinjiang from January 1st 2004–Dec 30th 2011. These sites were chosen due to their high HIV disease burden. Among them, Ruili, Longchuan, Lincang, Hezhou, Lingshan and Yining counties were ethnic minority areas. The Ethnic minority population accounted for 53.4% of the participants. Of these, Weiwuer (46.7%), Dai (23.1%), Jingpo (14.4%), Lisu and De’ang (8.0%) were the majorities. This study included all HIV pregnant women who received prenatal care, and follow-up started with the first clinic visit and up to 18 months after birth. Pregnant women lost to follow-up before the delivery or termination were excluded. As a result, we obtained HIV testing and status records of 221,217 pregnant women.

### Sampling and recruitment

HIV-infected pregnant women were followed up and managed by the local Women and Children’s hospital or antenatal care (ANC) clinics. ARV prophylaxis was provided by a team of doctors and nurses. HIV-infected pregnant women received a CD4 test before commencing ARV treatment. All experimental protocols were approved by the National Center for Women and Children’s Health, China Center for Disease Control (China CDC). All enrolled HIV-infected pregnant women have provided informed consent. Pregnant women were included if^1^ they were confirmed HIV positive^2^ and they chose to continue the pregnancy. However, pregnant women were excluded from the study if they were diagnosed with mental disease and were unable to participate in the study. A total of 1,426 HIV-infected pregnant women were enrolled in the study. A total of 1,387 participants continued the pregnancy and provided social and demographic information at the first ANC visit. Of 1,377 infants born to HIV-infected pregnant women, 10 were stillborn and 286 infants were lost to follow-up 3–12 months after birth.

### Treatment regimens for pregnant women and infants in China

All diagnosed HIV-infected pregnant women were referred to ART clinics. The treatment regimens were determined clinically (according to disease progression stages defined by the World Health Organization [WHO]) and/or immunologically (CD4 count or percentage). The 2004 national PMTCT guideline recommended HIV-positive pregnant women receive azidothymidine (AZT) twice a day during pregnancy. If diagnosed to be HIV-positive at delivery, pregnant women received AZT and one dose of nevirapine (NVP). HIV-exposed infants were provided with one dose of NVP and AZT every six hours up to six weeks postpartum. The 2008 national PMTCT guideline recommended that HIV-positive pregnant women should receive AZT twice a day from the 28^th^ week of gestation during pregnancy and at delivery, pregnant women receive AZT, lamivudine (3TC) and one dose of NVP. HIV-exposed infants were provided with one dose of NVP and AZT every six hours up to six weeks postpartum. The 2011 national PMTCT guidelines recommended changes to the 2004 guideline such that AZT+3TC+LPV/EFV (Lopinavir/Efavirenz) are used from 14 weeks of gestation twice daily. During delivery, pregnant women receive AZT+3TC and one dose NVP until seven days postpartum and HIV-exposed infants were offered one dose of NVP or AZT daily up to six weeks postpartum. Details have been previously described^16^. We regard treatment as ‘timely’ if HIV+ mothers were diagnosed and provided with ART before they reach the 28^th^ week of gestation.

### Data collection

By literature review, experts from various academic fields of maternal and children’s health, epidemiology, public health, anthropology, sociology, and psychology were invited to form a focus group and discuss the design of the questionnaire. The draft of the questionnaire was first piloted and revised based on feedback from participants and interviewers. The revised version was then used for the actual survey. Structured questionnaires were used in the study. The survey included sections on social characteristics, the progression of HIV, pregnancy history, sexual behaviors, HIV testing and time of testing, ARV regimens and time of ARV drug use, delivery method, the outcome of pregnancy and newborn, feeding mode and children’s HIV status. The survey was conducted by staff from the Women and Children’s hospitals. All HIV-infected pregnant women delivered in a hospital and were followed up by the staff of the participating hospitals accordingly. Follow-up took place at 42 days, 3, 6, 9, 12 and 18 months for both mother and child pairs. Infants were provided early diagnosis of HIV at study sites if the testing kits were available. All the children born to HIV-infected mothers were offered HIV antibody testing at 18 months.

Interviewers and translators received a standardised training program for the study. The identical questionnaire was used across eight study sites, and collected data was double entered and checked. Also, every three months, data from 5% of the respondents were randomly selected to check for inconsistencies and missing information.

### Data management and analysis

Data were entered and cleaned using Epidata version 3.5.1 and analyzed by SAS version 12.0 statistical package for Windows. A Cochran-Armitage trend test (χ^2^ statistics) was used to estimate the temporal changes of pregnant women undergoing HIV testing, the percentage of infant’s early diagnosis, 18-month follow-up, and the number of pregnant women and infants who received ARV according to the treatment guidelines. The likelihood of HIV vertical transmission was independently tested against the following factors: age, race, marital status, baseline CD4+ lymphocyte count, baseline HIV-1 RNA level; HIV-infected mothers using ARV regimen, infants born to HIV-infected mothers using ARV, regimen delivery method, feeding mode, sexual behavior, and others. A multivariate logistic model was used to determine predictors of mother to child transmission of HIV while adjusting for confounding factors. Variables with p < 0.10 in the univariate analyses were eligible for inclusion in the multivariate model. Variables were retained in the final model only if they were statistically significantly associated (p < 0.05) with the outcome or deemed to be of professional importance. A backward approach was used to select variables for the final model. The adjusted Odds Ratios (ORs) with its 95% confidence interval (CI) and p-values were used to measure the strength of association and identify a statistically significant result. A p-value < 0.05 was considered as a statistically significant association[Table t1].

### Ethical considerations

Ethical clearance was provided by the Institutional Review Board of the Center for National Women and Children’s Health, China Center for Disease Control. The study was conducted in accordance with China PMTCT guidelines 2011. All study protocols were approved by the Center for National Women and Children’s Health, China CDC. Informed consent was obtained from all participating mothers for blood samples collected.

## Results

### Demographic characteristics of pregnant women and newborns

During 2004–2011, a total of 221,217 pregnant women were covered by the program across the study sites and received HIV testing. Among them 1,491 women were found to be infected with HIV, accounting for 0.67% of the enrolled women. Of these HIV-infected pregnant women, 39 terminated their pregnancy and 65 did not wish to be in the PMTCT survey. These were excluded from further analysis. The remaining 1,387 participants provided social and demographic information at the first antenatal care clinic visit. Their average age was 27.9 years (range 16–45). Rural residents accounted for 74.9% (1039/1387), and 45.1% (626/1387) were ethnic Han. About 13.8% (192/1387) of participants had senior school or above education and 9.9% (137/1387) were illiterate. More than half of them (64.3% [748/1164]) were farmers. 27.3% (372/1361) HIV pregnant women were in their first pregnancy, and 46.4% (632/1363) had already had at least one child.

### Serological tests and delivery in PMTCT

**Figure 1:**
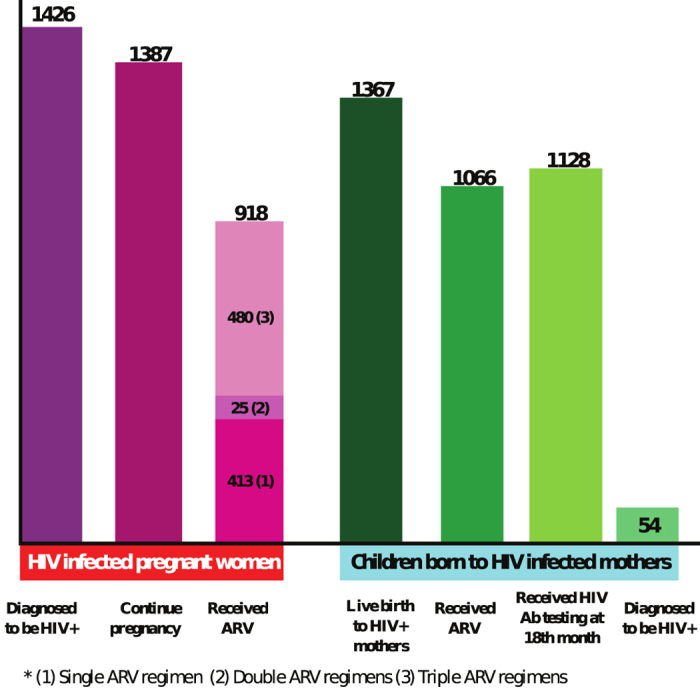
PMTCT continuum of care during 2004–2011 in China.

Among the 1,387 enrolled pregnant women, 298 received CD4+ T testing and 107 received virus load testing (Fig. 1). Only 1,005 pregnant women retained an accessible record of ARV usage. Among these, 918 received ARVs, and 187 gave birth without the use of ARVs. Of the women who received ARVs, 413, 25 and 480 used single, double and triple ARV regimens, respectively. Among these pregnant women, 120 (13.0%) commenced treatment before pregnancy; 386 (42.1%) started ARVs during pregnancy, and the rest, 412 (44.9%), received ARV intrapartum. Out of the 1,053 pregnant women for whom there was information recorded on delivery method, 61.3% (645/1053), 21.4% (225/1053) and 17.3% (183/1053) had a vaginal delivery, elective and emergency caesarean section, respectively. Three months after birth, 97.8% of infants received formula and the percentage increased slightly to 99.0% at six months (Fig. 1).

Of the 1,377 infants enrolled, only 1,091 infants retained ARVs usage records. Among these, 1,022 (93.7%) received ARV prophylaxis, and 939 (86.07%) received it with their mothers. Of the 1367 live births, 1,128 were followed up to 18 months and 1,124 received HIV antibody testing (Fig. 1). Of those who received HIV testing, 54 were diagnosed HIV positive, corresponding to an infection rate of 4.8% (Fig. 1). Infants of all 162 pregnant women received a viral load test and remained HIV negative. Average CD4+ cell count (/μl) of HIV-infected mothers over pregnancy did not affect infection status of their newborns.

### Improvement of PMTCT during 2004–2011

**Figure 2:**
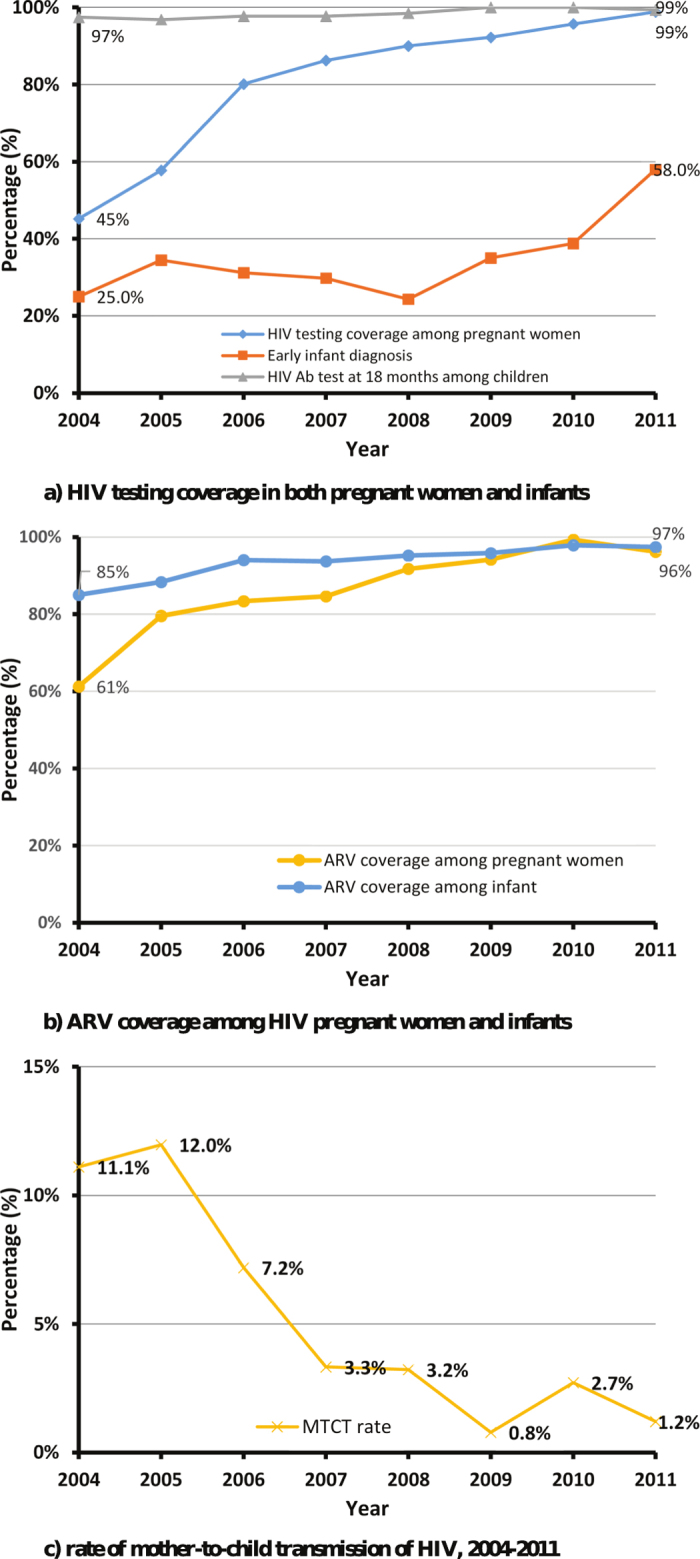
(**a**) HIV testing coverage in both pregnant women and infants. (**b**) ARV coverage among HIV pregnant women and infants. (**c**) Rate of mother-to-child transmission of HIV, 2004–2011.

**Figure 3:**
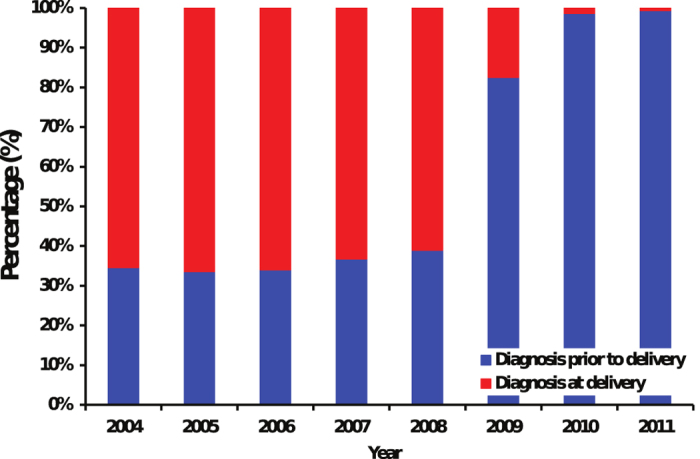
Diagnosis of HIV in infected mothers. Table 1 Status of mother-to-child transmission of HIV and associate factors.

Throughout the study period, HIV testing coverage increased in both pregnant women and infants (Fig. 2a). Among HIV-infected mothers, the proportion diagnosed before delivery varied from 30–40% during 2004–2008, but this percentage increased significantly to 83.5% in 2009 then to 98.9% in 2011 (Cochran-Armitage, Z = −17.17, p < 0.0001, Fig. 3). The percentage of HIV antibody testing at 18 months of the children born to HIV-infected mothers remained at a high level above 97% throughout the study period. The proportion of pregnant women receiving ARV prophylaxis increased from 61% in 2004 to 96% in 2011 (p < 0.0001). ARV coverage among children also showed a statistically significant increase from 85% in 2004 to 97% in 2011 (p < 0.0001, Fig. 2b). Notably, in 2004, only one pregnant woman received ARV prophylaxis during pregnancy whereas the majority (97.9%, 47) received single-dose NVP during delivery. This proportion has been dramatically reversed. In 2011, 93 (83.0%) pregnant women received ARV prophylaxis during pregnancy and only 9 (0.08%) received single-dose NVP during delivery (p < 0.0001). Among the 1,182 mother-child pairs followed up to 18 months after birth, the rate of mother-to-child transmission of HIV declined significantly from 11.1% in 2004 to 1.2% in 2011 (p < 0.0001, Fig. 2c).

### Factors associated with mother-to-child transmission of HIV

Regression analysis indicated that CD4+ T cell level, delivery method, feeding method, demographic and risk behaviors of the HIV-infected pregnant women were not statistically associated with vertical transmission of HIV. The risk of vertical transmission was significantly lower in infants born via vaginal delivery in comparison with emergency caesarean section (OR = 0.46; 95% CI [0.23–0.96]). HIV-infected mothers receiving multi-ARVs were also less likely to pass HIV to their infants (OR = 0.11; 95% CI [0.04–0.29]).

## Discussion

This study reported marked improvements in coverage of HIV testing, ARV and early use of ARV during pregnancy in pregnant women as a result of China’s PMTCT program. Our study indicated that the majority of HIV-infected women in our study adhere to the program during their pregnancy. In comparison, WHO estimated that in sub-Saharan Africa, only half of women living with HIV received any PMTCT intervention, 43% of HIV-exposed infants received ARV prophylaxis and merely 6–15% HIV-exposed infants received an HIV test in 2010^2^. We also reported a 10-fold decline in the mother-to-child transmission rate to 1.2% over the eight years studied. This rate is far lower than the national average in China (6.7%, [15]) and the millennium development goal (5% [5]). However, as this study was conducted in eight Chinese counties with a relatively severe HIV epidemic, it may not be representative of the national trend.

Multiple ARV prophylaxis during pregnancy was found to be a strong protective factor for mother-to-child transmission whereas vaginal delivery was also significantly associated with lower risk of mother-to-child transmission. Consistent results were found in previous studies in similar settings^17^,^18^. In 2011, in Malawi, where antiretroviral therapy (ART) was evaluated, only 4% of vertical transmission cases were detected within the first 48 weeks postpartum in the maternal ART arm^19^. Li *et al*., demonstrated that the risk of HIV vertical transmission is 4.4 times higher among mothers who received non-triple ARVs compared to those did receive^17^. Notably, our selected survey sites were located in high HIV disease burden areas, where there is higher awareness about mother-to-child transmission in both the government and the community^20^. The local PMTCT program has developed an effective service protocol based on the existing CDC antenatal care network to provide HIV care and encourage treatment compliance according to the guidelines. Early HIV detection during ANC visits and timely referral to ART sites have also enabled pregnant women to use ARV prophylaxis early. The vertical transmission rate reported in our study is far lower than that in areas where service provision was less effective (Guangzhou 13.89%; Shenzhen 5.3%^17^,^18^).

Timely diagnosis of HIV-infected mothers enables early intake of ARVs for their children, and this is reflected by the high percentage (97%) of HIV-exposed infants receiving ARVs in 2011.However, diagnosis of HIV-positive children remains a major gap, as only 52.4% infants received early infant diagnosis (EID 52.4%). We reported that 85% of exposed infants remained in care at 12–18 months after birth for the antibody screen test, and this is much higher than in other parts of China^21^,^22^. However, the remaining 15% still represents a missed opportunity for early diagnosis and intervention^23^,^24^. Similarly, issues of low ARV uptake and high drop-out rates among HIV-exposed infants after birth have also been reported in African settings (Kenya and Uganda)^25^,^26^. Improving the continuum of care for PMTCT is not only critical for preventing HIV in exposed infants but also for reducing mortality in infected infants. In 2014, EID was already piloted to be an integrated part of the PMTCT program in China, although it has yet to be expanded nationally. In the piloted areas, EID is routinely conducted among children born to HIV+ mothers during the same check-up time point for uninfected children. EID is expected to improve the survival rate among children infected with HIV by enabling them to be diagnosed and treated early. Further investment of resources and structural changes in the current ANC system are necessary for the future implementation of EID in China.

Interestingly, we reported a lower risk of HIV vertical transmission among mothers using vaginal delivery compared with those experiencing an emergency caesarean section delivery. This is consistent with previous findings^17^,^27^. Further, Briand *et al*. found that The MTCT rate did not differ according to the mode of delivery in term deliveries (≥37 gestational weeks), and his finding suggested that HIV-infected women on antiretroviral therapy have a very low HIV viral load and can safely opt for vaginal delivery in the absence of obstetrical risk factors^28^. Similar results were found by Townsend *et al*., that planned vaginal delivery does not contribute to a higher risk of vertical transmission than elective Cesarean section^29^. Similar findings have been reported by other related studies in China^17^,^18^,^30^,^31^. In our study and another study in Yunnan^31^, most HIV pregnant women were local residents, and adherence to interventions was shown to be better than migrant women^18^,^30^. There remain significant gaps in HIV testing coverage, ARVs utilization, and follow-up across geographical regions of China.

Several limitations of this study should be noted. First, this study is an observational study rather than an intervention study. The present study can only follow up patients enrolled in the program, and evaluation of the various ARV regimens cannot be conducted as a clinical trial. Second, limited availability of CD4+ T cell and virus load information limited the analysis of the association between ARV use and consequent immunological/virological impacts on mother-to-child transmission. These variables were not found to be statistically associated due to a substantial proportion of missing data. Third, the study has a long follow-up period. Participant adherence is crucial to the prevention of mother-to-child transmission of HIV. Those who are lost to follow-up often represent a subgroup of participants at higher risk of death or HIV infection. This may lead to underestimation of the actual HIV transmission rates in our study. Fourth, HIV status of the participants were collected retrospectively after delivery and the date of first-time HIV diagnosis was not clear except diagnoses at the time of delivery. We therefore could not stratify diagnoses according to pregnancy trimesters.

Despite the marked improvements in the PMTCT program outcomes in our study areas, we continue to observe missed opportunities in the continuum of care for PMTCT. This includes the failure to achieve a complete coverage of recommended ARVs regimens for HIV-infected women, early infant diagnosis, and substantial loss-to-follow-up among HIV-exposed infants. Nevertheless, our study highlights the importance of expanding HIV testing among pregnant women in their early stage of pregnancy, which ensures timely treatment for both mother and child. Providing a multi-ARV regimen or WHO Option B+ regimen at the ANC for all infected pregnant women should be recommended to reduce vertical transmission of HIV and maximize the overall health benefits for the mother-child pairs. Further research should focus on exploring the effectiveness of these interventions and the barriers to attending HIV care and treatment.

## Additional Information

**How to cite this article**: Wang, Q. *et al*. Timely antiretroviral prophylaxis during pregnancy effectively reduces HIV mother-to-child transmission in eight counties in China: a prospective study during 2004–2011. *Sci. Rep.* **6**, 34526; doi: 10.1038/srep34526 (2016).

## Figures and Tables

**Table 1 t1:** Regression analysis and factors associated with mother-to-child transmission of HIV.

Multivariate regression Indicators	Proportion (%)	Children HIV status			Univariate regression	Multivariate regression
		HIV+	HIV−	MTCT rate (%)	OR (95% CI)	OR (95% CI)
Demographic Characteristics						
Age						
<25	316 (28.6)	13	303	4.11	0.80 (0.31, 2.06)	
25~	408 (36.9)	21	387	5.15	1.02 (0.42, 2.44)	
30~	243 (22.0)	13	230	5.35	1.06 (0.41, 2.72)	
35~ (ref)	138 (12.5)	7	131	5.07		
Ethnic group						
Han	493 (46.6)	19	474	3.85	0.72 (0.40, 1.29)	
Minority (ref)	566 (53.4)	30	536	5.30		
Education						
Junior high and below (ref)	924 (87.3)	42	882	4.55		
Senior high and above	135 (12.7)	7	128	5.19	1.15 (0.51, 2.61)	
Marriage						
First	759 (72.2)	33	726	4.35	0.61 (0.14, 2.69)	
Second	264 (25.1)	14	250	5.30	0.76 (0.16, 3.51)	
Others (ref)	29 (2.7)	2	27	6.90		
Residence registration						
Rural (ref)	812 (76.7)	32	780	3.94		
Urban	247 (23.3)	17	230	6.88	1.80 (0.98, 3.30)	
Monthly income						
<500 RMB (ref)	573 (61.2)	25	548	4.36		
500–1000 RMB	246 (26.3)	17	229	6.91	1.63 (0.86, 3.07)	
>1000 RMB	117 (12.5)	3	114	2.56	0.58 (0.17, 1.94)	
HIV biomarkers and ARV use						
CD4						
>=350	264 (71.2)	5	259	1.89	2.61(1.17, 18.12)	
200–349	92 (24.8)	0	92	0	1.04 (0.33, 7.19)	
<200 (ref)	15 (4.0)	1	14	0.93		
Virusal load						
<50	57 (29.5)	0	57	0.00		
50–1000	27 (14.0)	0	27	0.00		
>1000 (ref)	109 (56.5)	0	109	0.00		
ARV use in HIV+ mothers						
Single regimen	413 (37.4)	25	388	6.05	0.51 (0.28, 0.94)	0.50 (0.25, 0.99)
Multi-regimen	505 (45.7)	8	497	1.58	0.13 (0.06, 0.29)	0.11 (0.04, 0.29)*
No use (ref)	187 (16.92)	21	166	11.23		
ARV use in HIV exposed children						
Yes	1006 (93.8)	45	961	4.47	0.47 (0.19, 1.14)	
No (ref)	66 (6.2)	6	60	9.09		
Delivery and feeding						
Delivery method						
Vaginal delivery	645 (61.3)	26	619	4.03	0.55 (0.28, 1.09)	0.46 (0.23, 0.96)*
Elective caesarean section	225 (1.4)	11	214	4.89	0.67 (0.29, 1.54)	0.74 (0.37, 2.03)
Emergency caesarean section (ref)	183 (174)	13	170	7.10		
Feeding mode						
Formula feeding	1033 (98.6)	49	984	4.74	0.20 (0.05, 0.73)	
Mixed feeding (ref)	15 (1.4)	3	12	20.00		
